# Relative microvessel area of the primary tumour, and not lymph node status, predicts the presence of bone marrow micrometastases detected by reverse transcriptase polymerase chain reaction in patients with clinically non-metastatic breast cancer

**DOI:** 10.1186/bcr980

**Published:** 2005-01-10

**Authors:** Ina H Benoy, Roberto Salgado, Hilde Elst, Peter Van Dam, Joost Weyler, Eric Van Marck, Simon Scharpé, Peter B Vermeulen, Luc Y Dirix

**Affiliations:** 1 Translational Cancer Research Group Antwerp (Laboratory of Pathology, University of Antwerp/University Hospital Antwerp, Edegem, Belgium, and Oncology Centre, General Hospital Sint-Augustinus, Wilrijk, Belgium); 2 Department of Epidemiology and Social Medicine, University of Antwerp, Wilrijk, Belgium; 3 Medical Biochemistry, University of Antwerp, Wilrijk, Belgium

**Keywords:** angiogenesis, bone marrow, breast cancer, Chalkley, micrometastasis.

## Abstract

About 50% of patients with breast cancer have no involvement of axillary lymph nodes at diagnosis and can be considered cured after primary locoregional treatment. However, about 20–30% will experience distant relapse. The group of patients at risk is not well characterised: recurrence is probably due to the establishment of micrometastases before treatment. Given the early steps of metastasis in which tumour cells interact with endothelial cells of blood vessels, and, given the independent prognostic value in breast cancer of both the quantification of tumour vascularisation and the detection of micrometastases in the bone marrow, the aim of this study was to determine the relationship between vascularisation, measured by Chalkley morphometry, and the bone marrow content of cytokeratin-19 (CK-19) mRNA, quantified by real-time reverse transcriptase polymerase chain reaction, in a series of 68 patients with localised untreated breast cancer. The blood concentration of factors involved in angiogenesis (interleukin-6 and vascular endothelial growth factor) and of factors involved in coagulation (D-dimer, fibrinogen, platelets) was also measured. When bone marrow CK-19 relative gene expression (RGE) was categorised according to the cut-off value of 0.77 (95th centile of control patients), 53% of the patients had an elevated CK-19 RGE. Patients with bone marrow micrometastases, on the basis of an elevated CK-19 RGE, had a mean Chalkley count of 7.5 ± 1.7 (median 7, standard error [SE] 0.30) compared with a mean Chalkley count of 6.5 ± 1.7 in other patients (median 6, SE 0.3) (Mann–Whitney *U*-test; *P *= 0.04). Multiple regression analysis revealed that Chalkley count, not lymph node status, independently predicted CK-19 RGE status (*P *= 0.04; odds ratio 1.38; 95% confidence interval 1.009–1.882). Blood parameters reflecting angiogenesis and coagulation were positively correlated with Chalkley count and/or CK-19 RGE. Our data are in support of an association between elevated relative microvessel area of the primary tumour and the presence of bone marrow micrometastases in breast cancer patients with operable disease, and corroborate the paracrine and endocrine role of interleukin-6 and the involvement of coagulation in breast cancer growth and metastasis.

## Introduction

The development of distant metastases is the primary cause of death in breast cancer patients. The involvement of the axillary lymph nodes, tumour size, histopathological grade and hormone receptor status determine prognosis and treatment options at initial diagnosis [1]. Nevertheless, these parameters do not accurately predict which patients will relapse after primary treatment, and they give limited information about the effectiveness of adjuvant treatment.

About 50% of patients have no involvement of the axillary lymph nodes at diagnosis and can therefore be considered cured after primary locoregional treatment. However, about 20–30% will experience distant relapse within 5–10 years, suggesting outgrowth of disseminated tumour cells present at diagnosis and undetectable by the current diagnostics [2]. This prompted the refinement of methods able to detect subclinical tumour deposits in various body compartments.

Tumour cells residing in bone marrow are considered to mirror the efficacy of the metastatic process throughout the body. Several prospectively designed clinical trials have confirmed the independent prognostic significance of the lodging of tumour cells in the bone marrow [3], suggesting that this minimal disease is indeed the progenitor of manifest metastasis. It is not clear whether the tumour cells that are part of subclinical metastases have arisen early during progression of the primary tumour or whether they are late and rare metastatic variants as a result of the cumulative acquisition of malignant phenotypic traits such as self-sufficiency in growth signals, insensitivity to anti-growth signals, evasion of apoptosis, limitless replicative potential, genomic instability, tissue invasion and sustained angiogenesis [4-6].

Bone marrow micrometastasis can be detected by immunocytochemical analyses with antibodies directed at epithelial markers. Polymerase chain reaction (PCR)-based techniques that amplify epithelial mRNA are more sensitive but need the introduction of cut-off values for positivity to correct for the inevitable loss of specificity.

The concept of dependence on vascularisation of growth, invasion and metastasis of malignant tumours has been challenged by the description of angiogenesis-independent mechanisms [7-10]. Nevertheless, the growth of most primary tumours needs angiogenesis. There is accumulating evidence that angiogenesis is intrinsically linked with the process of haemostasis [11]. Both angiogenesis and haemostasis are tightly regulated in physiological circumstances, for example during wound healing, but are deregulated when involved in tumour growth, invasion and metastasis. We have previously demonstrated the prognostic importance of the angiogenic cytokine interleukin (IL)-6, and the fibrin degradation product D-dimer, in patients with metastatic breast cancer [12,13].

A reproducible method of quantifying vascularisation, by assessing the relative microvessel area, is Chalkley point overlap morphometry [14]. Significant associations between the Chalkley count and axillary lymph node metastasis, increasing tumour size, high grade and histological subtype have been reported in patients with breast cancer. Moreover, an independent prognostic value has been demonstrated in patients with breast cancer by a meta-analysis of 87 published studies [15]. Chalkley counting is done in selected areas of high microvessel density, so-called 'hot spots'. The hypothetical rationale for counting in these highly vascular areas is that they predict the presence of more angiogenic subclones of the tumour [16]. These subclones might therefore have a higher metastatic efficiency.

The primary aim of this study was to assess the association of tumour vascularity, quantified by a well-standardised morphometrical method, and the presence of bone marrow micrometastases, detected by a sensitive and quantitative real-time reverse transcriptase PCR (RT–PCR) technique, in patients with operable breast cancer before treatment, to evaluate the relative microvessel area as a surrogate marker of bone marrow micrometastasis, as suggested by Fox and colleagues [17].

## Materials and methods

### Patients

Blood and bone marrow samples were collected preoperatively in 68 consecutive patients with localised, untreated, operable breast cancer. Eleven patients with a haematological malignancy who underwent bone marrow sampling for diagnostic purposes were entered as control patients for cytokeratin-19 (CK-19) relative gene expression (RGE).

### Tissue

A representative, full cross-section of the tumour sample surrounded by adjacent normal breast tissue was taken from all tumours. The tissue was fixed in buffered formalin and was paraffin-embedded. Sections 5 μm thick were cut and mounted on poly-L-lysine-coated slides.

### Blood coagulation tests

Plasma collection and measurement of fibrinogen, platelet counts and D-dimer levels were performed as described previously [12].

### Chalkley morphometry

Tumour vascularisation was evaluated by the Chalkley method, a morphometric point overlap counting system using a microscope eyepiece graticule. It has been shown to be both a rapid and a reproducible method that gives independent prognostic information and has been suggested as a standard method in an international consensus report on the quantification of angiogenesis [14]. In brief, a representative paraffin section including the tumour border was immunostained with a monoclonal anti-CD34 antibody (clone QBEnd/10; Biogenex, San Ramon, CA, USA) diluted 1:5 with overnight incubation at 4°C and without antigen retrieval procedure. The areas of highest vascular density ('hot spots') were identified at low magnification (× 10 ocular and × 10 objective). On a higher magnification (× 10 ocular and × 20 objective), a 25-point Chalkley eyepiece graticule (Chalkley grid area 0.22 mm^2^) was applied to each hot spot and orientated to permit the maximum number of points to hit on or in a microvessel. The Chalkley count in the hot spot with highest vascular density (the largest number of hits) was used for further analyses.

### Enzyme-linked immunosorbent assay (ELISA) of angiogenic cytokines

Serum samples were collected in serum separator tubes (type Vacutainer; Becton-Dickinson) and centrifuged at 2000 *g *for 5 min. Measurements of serum vascular endothelial growth factor (VEGF)-A_165 _and IL-6 were performed as described previously [18]. Plasma (Becton-Dickinson Vacutainer tubes type 9NC 0.129 M, 4.5 ml) VEGF-A_165 _was measured with an ELISA kit (R&D Systems, Minneapolis, MN, USA) on trisodium citrate-anticoagulated collected blood. For the ELISA assay of VEGF-A_165_, no cross-reactivity with other VEGF-A isoforms is documented. No cross-reactivity and no interference was found with IL-6-related (for example leukaemia inhibitory factor and oncostatin M) and growth factors not related to IL-6 (for example human platelet-derived growth factor and IL-7) (Quantikine human IL-6; R&D Systems, Minneapolis, MN, USA). Samples were assayed in duplicate. Within-assay variability had been tested before and was limited [18].

### Bone marrow sampling

Bone marrow (9 ml) was aspirated from the posterior iliac crest under local anaesthesia into syringes containing heparin as anticoagulant (Becton-Dickinson Vacutainer tubes type NH 170 IU 10 ml). Mononuclear cells were isolated by density-gradient centrifugation through Ficoll-Paque (Amersham Pharmacia Biotech) and washed twice with phosphate-buffered saline. After final centrifugation, the cell pellets were resuspended in a guanidine-containing buffer and stored at -70°C.

### Cell line

The MDA-MB 361 cell line (American Type Culture Collection [ATCC] catalogue code HTB-27) was cultured in Leibovitz's L-15 medium with a free gas exchange with atmospheric air, and supplemented with 20% fetal bovine serum and antibiotics. The medium was replaced every 3–4 days. Cells were harvested in accordance with the ATCC guidelines.

### RNA isolation and cDNA synthesis

Total RNA was extracted from the mononuclear cell fraction with the RNeasy kit (Qiagen). The amount of RNA was measured spectrophotometrically. Only samples with an *A*_260_/*A*_280 _ratio of more than 1.8, indicating high purity, were used. The RNA integrity was tested with the Agilent Bioanalyzer (Agilent Technologies). Only samples with a lack of degradation on the electrophoretogram and with a 28S/18S ratio of at least 1.9 were analysed. For first cDNA strand generation, 2 μg of total RNA was reverse-transcribed with the High-Capacity cDNA Archive kit (Applied Biosystems) in a total volume of 100 μl.

### Primers and probe design

Primers and probe for CK-19 were designed with the aid of Primer Express software (Applied Biosystems). To avoid amplification of contaminating genomic DNA, primers and probe were located on different exons.

The forward primer of CK-19 (5'-CCCGCGACTACAGCCACTA-3') is situated on exon 1, the probe (5'-ACCATTGAGAACTCCAGGATTGTCCTGCA-3', labelled with 6-carboxyfluorescein [FAM] at the 5' end and 6-carboxy-tetramethylrhodamine [TAMRA] at the 3' end) on exon 2 and the reverse primer (5'-CTCATGCGCAGAGCCTGTT-3') on exon 3. RT-PCR with this primer set resulted in a 163-base-pair fragment. The nucleotide sequences of the primers and probe were checked for their specificity in the NCI BLAST^® ^database. The amplification product was sequenced and was comparable with the predicted nucleotide sequence.

### PCR amplification

All PCR reactions were performed on the ABI Prism 7700 Sequence Detection System (Applied Biosystems) with the fluorescent Taqman method. Mispriming of processed pseudogenes was excluded.

The CK-19 mRNA quantities were analysed in triplicate, normalised against β-actin as a control gene and were expressed in relation to a calibrator sample. The calibrator was produced from the blood of a healthy volunteer spiked with five MDA-MB 361 cells per 10^6 ^mononuclear cells. The calibrator was given a RGE of 100. As described by Livak and colleagues [19], results are expressed as RGE with the ΔΔ*C*_T _method.

### Ethical considerations

The Ethical Committee of both institutions approved this study. Written informed consent was obtained from all patients.

### Statistical analysis

Statistical analysis was performed with Graphpad Prism (version 2.0; Graphpad Software, Inc.). For IL-6 and VEGF-A ELISA assays, half of the detection limit value of the patient samples was used for statistical analysis in case the measured values did not reach the detection limit of the assay. Detection limits were 0.7 and 9 pg/ml, respectively. Comparisons of continuous variables were performed with the Mann–Whitney *U*-test. Relationship between categorical variables was validated with a χ^2 ^test. The correlation of continuous variables was analysed with a Spearman rank test. A multiple regression analysis was performed with a stepwise-backward likelihood procedure. *P *< 0.05 was necessary for statistical significance.

## Results

### Patients

Age, T status, N status, oestrogen and progesterone receptor-status, tumour differentiation, menopausal status and tumour histology are given in Table 1.

### CK-19 RGE

Quantification of the mRNA transcripts of CK-19 in the bone marrow aspirates could be performed in all control samples and in 62 bone marrow samples of patients with breast cancer. In six patients, the volume of bone marrow or the quality of RNA was insufficient. A median RGE of 0.57 (range 0.22–0.78) was found for the control samples. Patients with primary breast cancer had a mean RGE of 3.85 ± 20.95 (median 0.79, standard error [SE] 2.66). Values above the 95th centile of the control values (0.77) were considered to indicate the presence of bone marrow micrometastasis. With this cut-off, 33 of 62 patients (53%) had bone marrow micrometastasis. In 49% of patients there was a concordance between lymph node status and bone marrow status. Fifty-five percent of patients with bone marrow micrometastasis had negative lymph nodes. Forty-six percent of patients without bone marrow micrometastasis had positive lymph nodes. Fifty-five percent of patients without lymph node metastasis had bone marrow micrometastasis. Forty-six percent of patients with lymph node metastasis had no bone marrow micrometastases.

### Chalkley count

Quantification of relative microvessel area by Chalkley morphometry was performed in all (*n *= 68) breast cancer samples. A mean value of 7.1 ± 1.9 (median 7, SE 0.23) was found. The median value of 7 was taken as a cut-off for categorisation into 'high' (7 or more) and 'low' Chalkley count. There was a positive association of Chalkley count with T-stage, but not with lymph node status (Table 2).

### CK-19 RGE and Chalkley count

**Figure 1:**
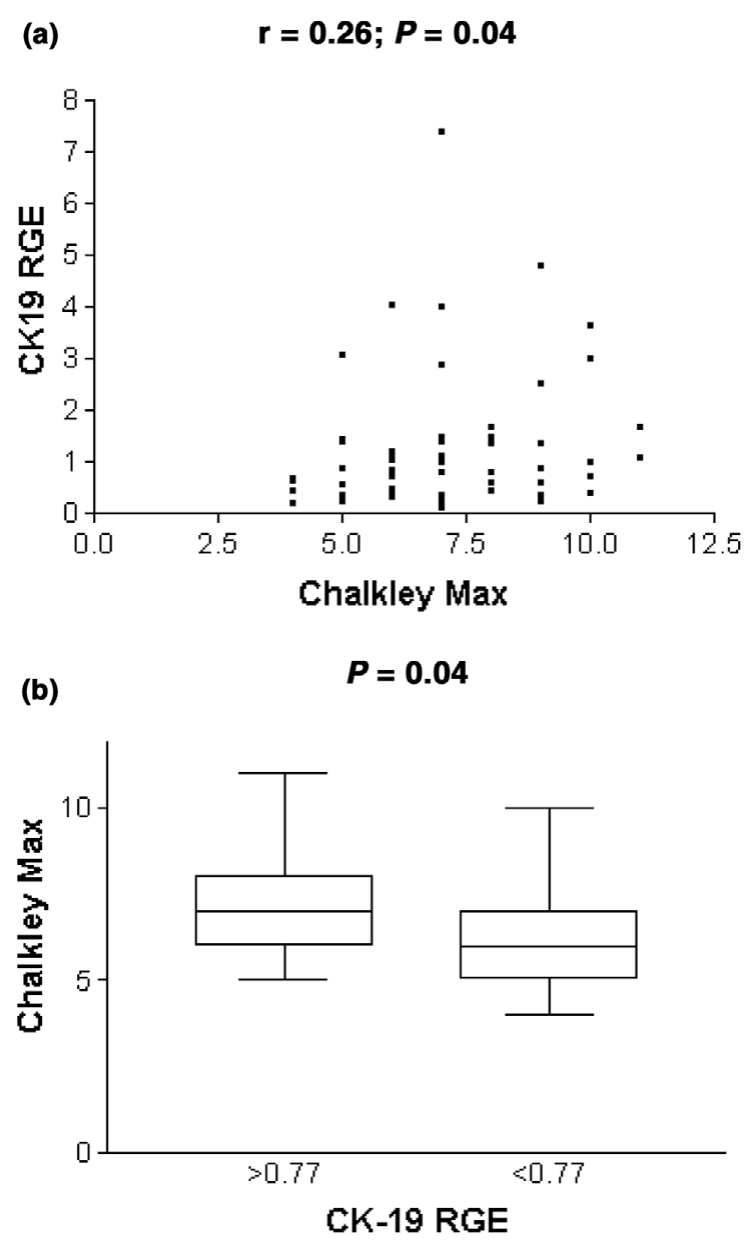
Chalkley count and relative gene expression of cytokeratin-19 (CK-19 RGE). **(a) **Correlation analysis of Chalkley count and CK-19 RGE. **(b) **Chalkley counts of patients with and without bone marrow metastases (according to the cut-off value of 0.77 for CK-19 RGE). Box-and-whisker plot limits depict 75th and 25th centiles and median value (box), and upper/lower quantile ± 1.5 × (interquantile range) (upper and lower whiskers, respectively).

Considering both Chalkley counts and CK-19 RGE as continuous variables, a significant correlation was found (*r *= 0.26, *P *= 0.04; Fig. 1a).

Sixty-two percent (23 of 37 patients) of breast tumours with a high Chalkley count were related to a bone marrow sample with positive CK-19 RGE. This was true for only 40% (10 of 25 patients) of breast tumours with a low Chalkley count (χ^2 ^test *P *= 0.04).

Patients with a CK-19 RGE of 0.77 or more had a tumour with a mean Chalkley count of 7.5 ± 1.7 (median 7.0, SE 0.3) compared with 6.5 ± 1.7 (median 6.0, SE 0.3) in patients with a CK-19 RGE of less than 0.77 (Mann–Whitney *U*-test *P *= 0.04; Fig. 1b).

A stepwise-backward likelihood multiple regression analysis for the prediction of the presence of bone marrow micrometastases was performed, including Chalkley count, serum IL-6, D-dimers, fibrinogen, platelet count, serum VEGF-A, plasma VEGF-A, T-stage, N-stage, oestrogen receptor status, progesterone receptor status, tumour type and differentiation status. Only Chalkley count, as a continuous variable, independently predicted CK-19 RGE status (*P *= 0.04; odds ratio 1.38; 95% confidence interval 1.009–1.882).

### Coagulation parameters and angiogenesis parameters in blood

Values and associations are given in Tables 3 and 4. In general, parameters reflecting coagulation and angiogenesis in blood had positive significant associations.

### Chalkley count, coagulation parameters and angiogenesis parameters in blood

**Figure 2:**
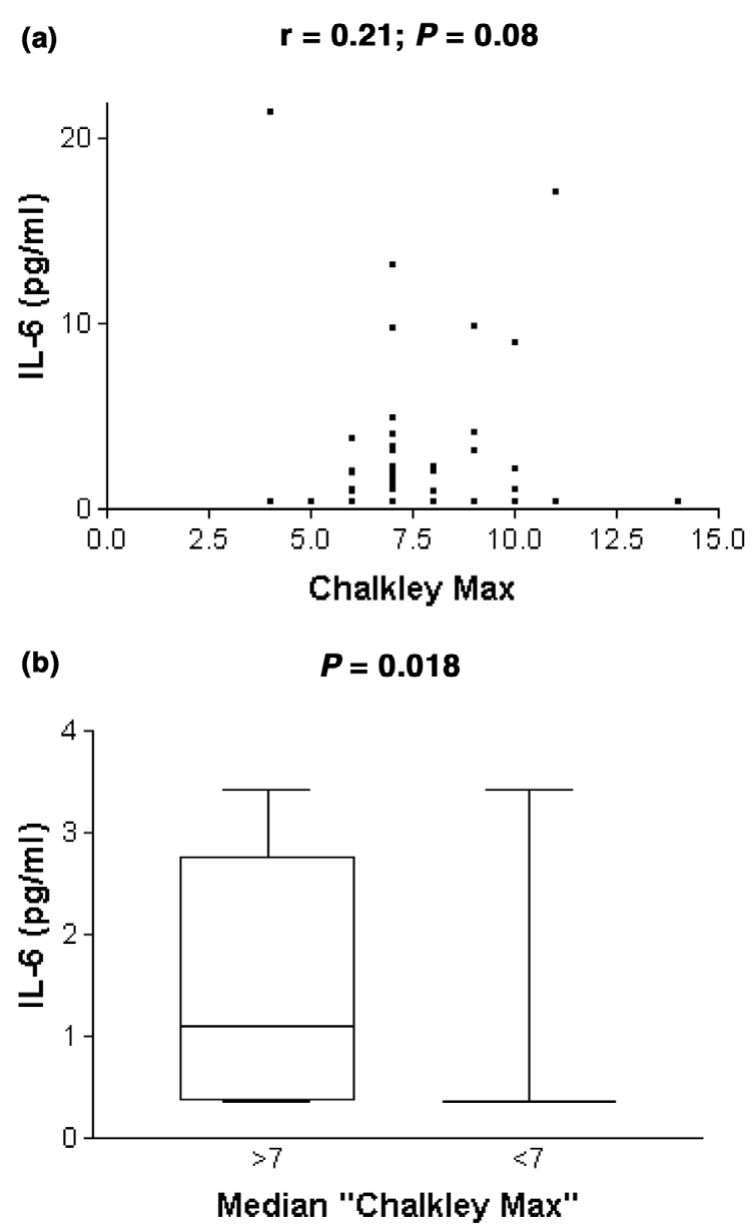
Chalkley count and interleukin-6 (IL-6). **(a) **Correlation of IL-6 serum levels and Chalkley count. **(b) **IL-6 serum levels of patients with a tumour with high and low Chalkley count (according to the cut-off value of 7).

**Figure 3:**
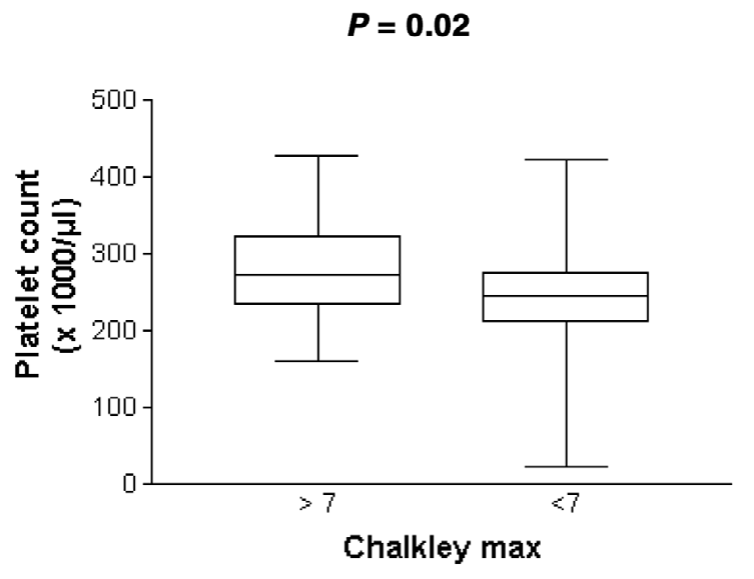
Analysis of platelet count in relation to high and low Chalkley count (according to the cut-off value of 7).

Serum IL-6 levels were correlated with Chalkley count (*r *= 0.2, *P *= 0.08; Fig. 2a). The mean IL-6 concentration in the serum of patients with a high Chalkley count was 2.57 ± 3.75 pg/ml (median 1.10, SE 0.57), and 1.52 ± 4.21 pg/ml (median 0.35, SE 0.84; Mann–Whitney *U*-test *P *= 0.018; Fig. 2b) in patients with a low Chalkley count. Platelet count in patients with a high Chalkley count was (282.8 ± 64.6) × 10^3^/μl (median 272, SE 9.8), and (240 ± 73.9) × 10^3^/μl (median 243, SE 14.8; Mann–Whitney *U*-test *P *= 0.02; Fig. 3) in patients with a low Chalkley count.

### CK-19 RGE, coagulation parameters and angiogenesis parameters in blood

**Figure 4:**
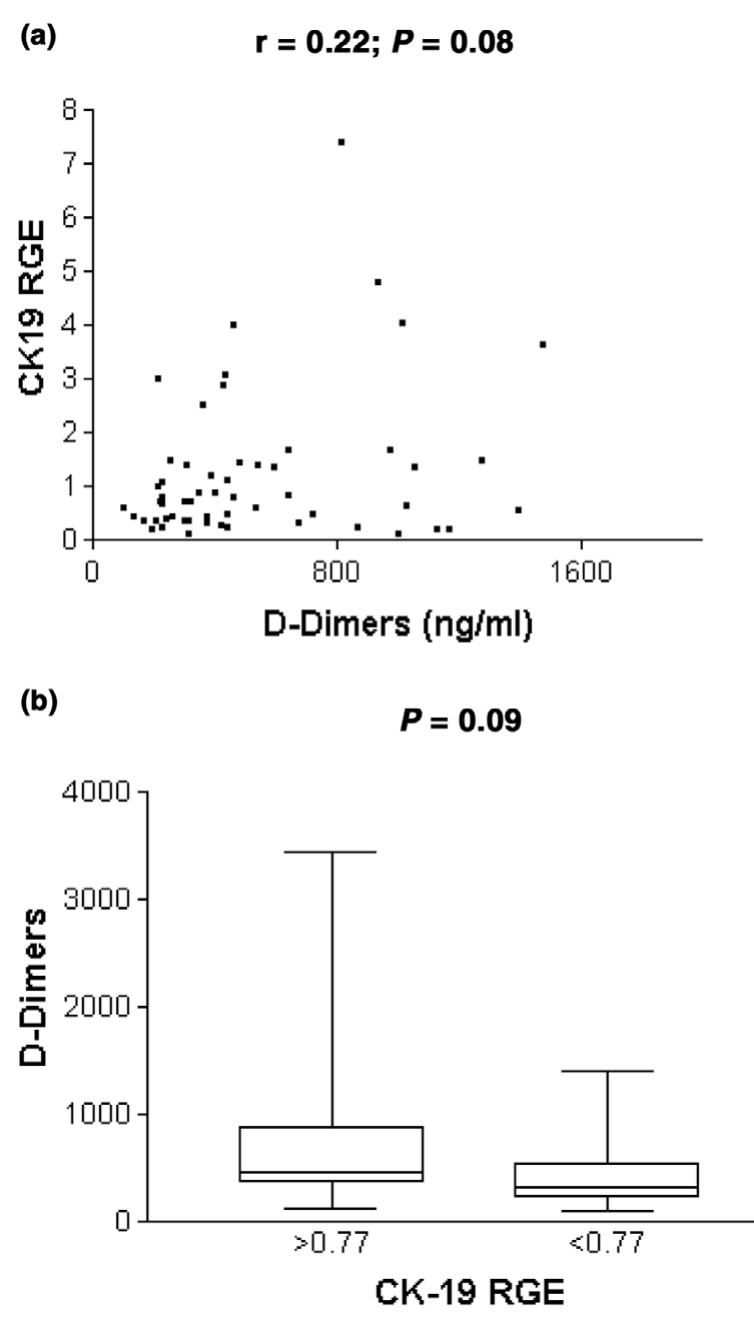
Cytokeratin-19 relative gene expression (CK-19 RGE) and D-dimers. **(a) **Correlation analysis of CK-19 RGE and D-dimers. **(b) **Analysis of D-dimers in relation to categorised CK-19 RGE.

D-dimer levels were correlated (*r *= 0.22, *P *= 0.08; Fig. 4a) with CK-19 RGE. Forty-one patients had elevated (more than 250 ng/ml) D-dimer levels. A mean D-dimer concentration of 477 ± 362 ng/ml (median 319, SE 67) was found in patients with CK-19 RGE values of less than 0.77, whereas the mean D-dimer level was 674 ± 634 ng/ml in patients with a CK-19 RGE value of 0.77 or more (median 458.5, SE 115.8; Mann–Whitney *U*-test *P *= 0.09; Fig. 4b). No associations between CK-19 RGE and other variables were found (data not shown).

## Discussion

The relation between the relative microvessel area, quantified by Chalkley counting of immunostained blood vessels in sections of primary breast cancer, and the pre-operative quantification of micrometastases in bone marrow by Taqman real-time RT–PCR was investigated in a group of patients with primary breast cancer before the start of treatment. Every increase in Chalkley count independently predicted a higher likelihood of bone marrow epithelial cells (odds ratio of 1.4) in patients with non-metastatic breast cancer. Patients with RGE of CK-19 in the bone marrow above the 95th centile of a control population had a primary tumour with a Chalkley count of 7.5, compared with 6.5 in patients without PCR-detected bone marrow micrometastases (*P *= 0.04).

Our work confirms the study by Fox and colleagues [17]. In 214 patients with primary breast cancer, tumour cells were detected through the examination of epithelial membrane antigen expression and the analysis of cell morphology. In immunostained breast cancer sections, a semi-quantitative vascular grade, expressed as being low or high, was determined in so-called vascular hot spots. Absolute concordance by kappa statistics of this vascular grade and Chalkley count was reported in a small subset of 22 patients. High vascular grade and the presence of vascular invasion independently predicted the presence of bone marrow micrometastases (respective odds ratios 2.7 and 2.7). In our study, Chalkley counts were determined in all 68 patients. However, the main difference lies in the higher sensitivity of Taqman quantitative real-time RT–PCR than that of immunocytochemistry on bone marrow cytospins used by Fox and colleagues [17].

This study corroborates the well-documented prognostic value of the Chalkley count in breast cancer [20]. Hansen and colleagues [20] categorised Chalkley counts by using cut-off points of 5 and 7. Patients with Chalkley counts of 7 or more had a hazard ratio for death of 1.46 (95% confidence interval 1.14–1.87) compared with the group of patients with Chalkley counts between 5 and 7. Bone marrow micrometastases are predicted by a high Chalkley count ([17] and this study) and have been linked to adverse outcome in patients with breast cancer: currently, about 2500 patients with breast cancer have been analysed in five prospectively designed clinical trials to verify associations between the presence of immunostained tumour cells in bone marrow and prognosis [3]. Braun and colleagues [21] have included 552 patients with stage I, II and III breast cancer in a prospective study: the presence of tumour cells detected with the antibody A45-B/B3 independently predicted distant metastasis but not locoregional recurrence. The association of a high Chalkley count with the presence of tumour cells in the bone marrow explains at least partly the prognostic value of the former parameter.

The presence or absence of lymph node metastases did not predict the bone marrow CK-19 RGE status by multiple regression analysis in this study. Concordance between bone marrow status and lymph node status was present in only 49% of all patients. This is in accord with results of cDNA array analyses of primary breast tumours of bone marrow-positive and bone marrow-negative patients [22]. Distinct profiles were reported, indicating that bone marrow metastasis is a selective process with a specific molecular signature of the primary breast tumour. In addition, in a prognostic study of patients with operable breast cancer, the bone marrow status provided independent information in addition to tumour size and lymph node status in a multivariate analysis [23]. The presence of bone marrow micrometastases was related to earlier recurrence.

Although we did not compare the CK-19 RGE with the number of immunostained tumour cells in bone marrow cytospins in this study, the Taqman quantitative real-time RT–PCR methodology has been validated by us in a group of patients with metastatic breast cancer [24]. RT–PCR quantification of mRNA transcripts of epithelial cells has a high sensitivity but a rather poor specificity for the detection of tumour cells in bone marrow or blood. The main reason for this is the expression of some epithelial markers, such as CK-19, by haematopoietic and blood cells. A stringent cut-off for CK-19 RGE positivity has therefore been set in this study. Methodological adaptations to avoid mispriming due to pseudogenes and to avoid amplification of contaminating DNA have also been made. Expression of the CK-19 RGE as an absolute number of tumour cells was not performed, because of unpredictable variations of the expression levels of CK-19 mRNA in tumour cells from individual patients.

What are the putative tumour-biological mechanisms to explain the positive association between the extent of tumour vascularisation and the amount of disseminated epithelial cells, and thus probably to a large extent 'tumour' cells, in the bone marrow of breast cancer patients? A first explanation might be that intravasation of breast cancer cells, one of the initial steps of metastasis, is facilitated in tumours with extensive vascularisation [25-27]. McCulloch and colleagues [28] indeed demonstrated a positive association between, on the one hand, the number of tumour cells in the blood of the veins draining the breast gland during surgery and, on the other, the vascularity of the primary tumour [28,29]. Although mechanical stress due to diagnostic procedures, such as mammography, or due to surgery itself can augment tumour cell shedding, this study links high vascularity to increased intravasation of breast cancer cells in a peri-operative setting. A comparable positive relation between breast tumour vascularity and CK-19 mRNA in the blood was found in patients 2–3 weeks after surgery [30]. An increased endothelial surface area might provide easier access to the bloodstream. A complementary effect of high vascularity is the more pronounced paracrine interaction between endothelial cells and tumour cells. Activated endothelial cells can indeed produce tumour cell growth factors, for example IL-6 in the vertical growth phase of melanoma [31], or matrix remodelling enzymes that facilitate tumour cell motility and invasion, as demonstrated in skin carcinogenesis models [32]. The survival and growth of tumour cells can also be influenced by endocrine interactions with a primary tumour present. In cervical, ovarian and colorectal cancer IL-6 is shed in the blood by the tumour, creating a 2.5-fold arteriovenous gradient [33]. In this study, patients with a Chalkley count of 7 or more had concentrations of circulating IL-6 that were about 2.5-fold higher than patients with a Chalkley count of less than 7 (*P *= 0.018). This is in accord with the angiogenic potential of IL-6. In metastatic breast cancer, high levels of circulating IL-6 predict a shortened survival time [13].

Unlimited haemostasis is intimately linked to tumour growth and angiogenesis, and tumours can therefore be regarded as non-healing wounds [34]. Immunohistochemical analyses with antibodies directed at fibrin demonstrated abundant stromal fibrin depositions in inflammatory breast cancer, a highly aggressive and highly angiogenic type of breast cancer, and less or no fibrin in non-inflammatory breast cancer [35]. A relation of high Chalkley counts and the presence of fibrin in the tumour was also reported in cutaneous breast cancer deposits [36]. In this study, levels of D-dimers in plasma, as a reflection of active fibrin remodelling, were correlated with CK-19 RGE values (*r *= 0.23; *P *= 0.08). Levels of D-dimers in patients with CK-19 RGE values of 0.77 or more were 1.4-fold those in patients with CK-19 RGE values of less than 0.77 (*P *= 0.09). Significantly higher levels of plasma D-dimers have been found in veins draining colorectal adenocarcinomas than in arteries [33], suggesting that plasma D-dimer levels are a measure of matrix remodelling in the tumour. Elevated levels of circulating D-dimers have been correlated with enhanced progression kinetics and reduced overall survival in metastatic breast cancer [12]. Taken together, these results strongly suggest interactions between angiogenesis and haemostasis to facilitate metastasis in breast cancer.

## Conclusion

In conclusion, our data show that tumour vascularity, and not lymph node status, independently predicts the presence of PCR-detected bone marrow micrometastases. This is consistent with direct haematogenous spread from the primary breast cancer without orderly progression from local to nodal to distant disease.

## Abbreviations

CK = cytokeratin; ELISA = enzyme-linked immunosorbent assay; IL = interleukin; RGE = relative gene expression; RT–PCR = reverse transcriptase polymerase chain reaction; SE = standard error; VEGF = vascular endothelial growth factor.

## Competing interests

The author(s) declare that they have no competing interests.

## Authors' contributions

IB performed the RT–PCR reactions, the blood coagulation tests and the ELISA analysis of the angiogenic cytokines. She made substantial contributions to analysis and interpretation of data, and drafted the manuscript. RS performed the Chalkley morphometry and made substantial contributions to analysis and interpretation of data, and drafted the manuscript. HE performed the cell line culture, the primers and probe design and optimised the RT–PCR reaction. PvD, EVM and SS made substantial contributions to conception and design. JW performed the statistical analysis. PV conceived of the study and has been involved in drafting the article and revising it critically for important intellectual content. LD conceived of the study and has given final approval of the version to be published. All authors read and approved the final manuscript.

## Figures and Tables

**Table 1:** Clinicopathological characteristics of patients and tumours (*N *= 68)

Characteristic	Value
Mean age in years (SD)	60.6 (12.3)
T status	
T1	30
T2	15
T3	13
T4	10
N status	
N0	36
N1	28
N2	4
Hormone receptor status	
ER^+ ^and/or PgR^+^	59
ER^-^/PgR^-^	9
Tumour differentiation	
I	27
II	27
III	14
Menopausal status	
Pre- and perimenopausal	11
Postmenopausal	57
Histology	
IDA	59
ILA	8
Medullar	1

ER, oestrogen receptor; IDA, invasive ductal adenocarcinoma; ILA, invasive lobular adenocarcinoma; PR, progesteronereceptor; SD, standard deviation.

**Table 2:** Contingency table comparing vascularisation (Chalkley count: low [less than 7] and high [7 or more]) with the clinicopathological characteristics of the patients with breast cancer (χ^2 ^test *P *value)

Characteristic	Vascularisation		*P*
		
	Low	High	
Age in years			
<50	4 (40%)	6 (60%)	0.97
50–64	12 (36%)	21 (64%)	
>65	9 (36%)	16 (64%)	
Lymph node status			
Negative	16 (44%)	20 (56%)	0.26
Positive	10 (31%)	22 (69%)	
Grade			
I	10 (37%)	17 (63%)	0.79
II	9 (33%)	18 (67%)	
III	6 (43%)	8 (57%)	
Oestrogen receptor			
Negative	17 (34%)	33 (66%)	0.43
Positive	8 (44%)	10 (56%)	
Micrometastases (CK-19 RGE)			
≥ 0.77	10 (30%)	23 (70%)	0.04
<0.77	15 (52%)	14 (48%)	
T-stage			
T1/T2	21 (47%)	24 (53%)	0.05
T3/T4	5 (22%)	18 (78%)	

CK-19 RGE, cytokeratin-19 relative gene expression.

**Table 3:** Coagulation and angiogenesis factors

Factor	*N*	Mean (median)	SD (SE)	95% CI
sIL-6	68	2.18 (0.35)	3.92 (0.48)	1.21–3.13
sVEGF	68	356.4 (285.9)	259.5 (31.47)	293.5–419.2
pVEGF	67	34.81 (22.2)	52.56 (6.42)	21.99–47.63
D-D	63	570.1 (419)	504.9 (63.61)	442.9–697.2
Platelets	68	267.2 (266)	70.66 (8.57)	250.1–284.3
Fibrinogen	63	320.9 (306)	73.47 (9.26)	302.4–339.4
sVEGF/pl	68	1.37 (1.15)	0.92 (0.11)	1.15–1.60

Abbreviations and units: D-D, D-dimers (ng/ml); platelets, 10^3 ^platelets/μl; pVEGF, plasma vascular endothelial growth factor (pg/ml); sIL-6, serum interleukin-6 (pg/ml); sVEGF, serum VEGF (pg/ml); sVEGF/pl, serum VEGF (pg/ml) per 10^6 ^platelets. Fibrinogen is shown in mg/dl. CI, confidence interval; SD, standard deviation; SE, standard error.

**Table 4:** Correlation of coagulation and angiogenesis markers

	*r*/*P*						
	
	sIL-6	sVEGF	pVEGF	D-D	Platelets	Fibrinogen	sVEGF/pl
sIL-6		0.25/0.03	0.33/0.005	0.41/0.006		0.23/0.06	0.22/0.06
sVEGF			0.48/<0.001	0.25/0.04	0.22/0.06		0.88/<0.0001
pVEGF				0.38/0.0019	0.23/0.05	0.26/0.0339	0.38/0.001
D-D						0.26/0.03	0.34/0.0061

D-D, D-dimers; pVEGF, plasma vascular endothelial growth factor; sIL-6, serum interleukin-6; sVEGF, serum VEGF; sVEGF/pl, serum VEGF per 10^6 ^platelets. Spearman correlation test *r *values and *P *values (*r*/*P*) are given if there was a statistical trend or a significant difference.
